# Operative technique at caesarean delivery and risk of complete uterine rupture in a subsequent trial of labour at term. A registry case-control study

**DOI:** 10.1371/journal.pone.0187850

**Published:** 2017-11-14

**Authors:** Dorthe L. A. Thisted, Laust H. Mortensen, Lone Hvidman, Lone Krebs

**Affiliations:** 1 Fetal Medicine Unit. Department of Gynaecology and Obstetrics, University of Copenhagen, Hvidovre Hospital, Kettegaard Alle 30, Hvidovre, Denmark; 2 Methods and Analysis, Statistics Denmark, Sejeroegade 11, Copenhagen, Denmark; 3 Section of Social Medicine, University of Copenhagen, Oester Farimagsgade Copenhagen, Denmark; 4 Department of Obstetrics and Gynaecology Aarhus University Hospital, Skejby, Aarhus, Denmark; 5 Department of Gynaecology and Obstetrics, University of Copenhagen, Holbaek Hospital, Holbaek, Denmark; Univesity of Iowa, UNITED STATES

## Abstract

**Objective:**

To estimate the relation of single-layer closure at previous caesarean delivery, and other pre-labour and intra-partum risk factors for complete uterine rupture in trial of vaginal birth after a caesarean (TOLAC) at term.

**Study design:**

Population-based case-control study. We identified all women (n = 39 742) recorded in the Danish Medical Birth Registry (DMBR) during a 12-year period (1997–2008) with a singleton pregnancy at term and TOLAC. Among these, all women with a complete uterine rupture were identified (cases). Information from the registry was validated against medical records. Controls were selected in the DMBR as the following two births with TOLAC at term and no uterine rupture. Detailed information from cases and controls was collected from manual review of medical records. Main outcome measure was **c**omplete uterine rupture during TOLAC at term.

**Results:**

Upon validation, 175 cases and 272 controls met the above criteria. After adjustment for possible confounding factors there was no association between single layer closure and uterine rupture (aOR 1.38, CI: 0.88–2.17). Significant risk factors were: Induction with an unfavourable cervix (aOR 2.10 CI: 1.19–3.71), epidural (aOR 2.17 CI 1.31–3.57), augmentation by oxytocin for more than one hour (aOR 2.03 CI: 1.20–3.44), and birth weight ≥ 4000g (aOR 2.65 CI 1.05–6.64). Previous vaginal delivery (aOR 0.41 CI: 0.25–0.68) and inter-delivery interval of more than 24 months (aOR 0.38 CI: 0.18–0.78) reduced the risk of uterine rupture.

**Conclusion:**

Single-layer uterine closure did not remain significantly associated to uterine rupture during TOLAC at term after adjustment for confounding factors. Induction of labour with an unfavourable cervix, birth weight ≥ 4000g and indicators of prolonged labour were all major risk factors for uterine rupture.

## Introduction

Rupture of the pregnant uterus almost exclusively occurs among women who attempt a trial of labour after a caesarean delivery (TOLAC) [1]. A complete uterine rupture is associated with a very high perinatal mortality and a substantial perinatal and maternal morbidity. The complete uterine rupture is uniquely described as a complete rupture of the myometrium and rupture of the fetal membranes leading to a direct communication between the uterine cavity and the peritoneum [2–5]. The incidence of complete uterine rupture in TOLAC in high-income countries varies from 0.22% to 0.74% [6–8].

In 1998, the Danish Society of Obstetrics and Gynaecology recommended a new operative technique for caesarean delivery. The most important changes was to suture the uterus in a single layer, instead of two layers as previously done and to leave the visceral and parietal peritoneal layers of peritoneum open [9].

Many studies have investigated the impact of single layer closure and the risk of uterine rupture in TOLAC. Results have been conflicting, and given the rare occurrence of uterine rupture, many studies have been underpowered [10]. Other studies included both complete and partial uterine ruptures [1,10–15].

The primary aim of this study was to estimate the risk of complete uterine rupture in singleton term pregnancies attempting a TOLAC when the uterus was initially closed in one layer compared to two layers in a previous caesarean delivery. The secondary aim of the study was to identify other possible risk factors for complete uterine rupture related to the actual TOLAC.

## Materials and methods

The Danish Medical Birth Registry (DMBR) contains data on all deliveries in Denmark [16].

Pre-pregnancy risk factors, medical diseases, and complications and interventions during pregnancy and delivery are recorded by codes according to the International Classification of Diseases and Related Health Problems 10th Revision [17] and the Nordic Medico-statistical Committee classification of surgical procedures [18]. A 10-digit personal identification is assigned to all Danish citizens, making it possible to link registries and medical records.

This study was based on data from the DMBR from January 1, 1997 to December 31, 2008. During the study period, 705,871 women had a singleton birth at hospital, of these 62,475 women had previous caesarean delivery (8.85%), and among these 39,472 (63.2%) attempted a vaginal delivery.

**Fig 1:**
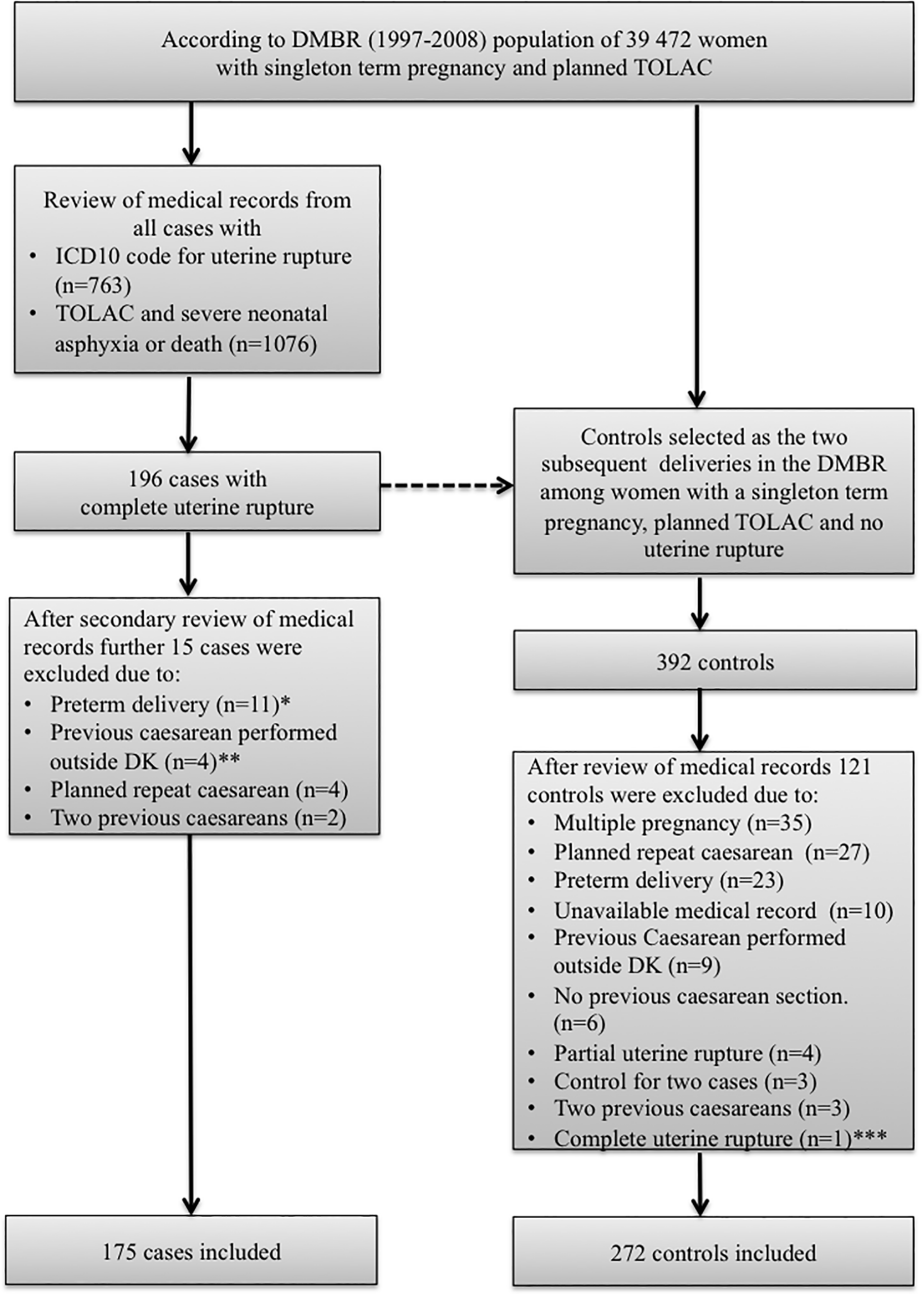
Selection of cases and controls. *Gestational age < 37+0 weeks. ** Previous caesarean section were performed outside Denmark, we were not able to exclude that a classical caesarean section were performed ***Were among cases. DMBR Danish Medical Birth Registry. TOLAC Trial of labour after caesarean.

Cases were identified in the DMBR (1997–2008) among all women with a singleton term pregnancy, with TOLAC, who were recorded in the DMBR with uterine rupture during labour (n = 763). A complete uterine rupture is associated with a high perinatal mortality and morbidity, and knowing that the recording of uterine rupture in the DMBR may be incomplete, we furthermore identified all cases (n = 1076) with TOLAC and where the perinatal outcome was severe perinatal asphyxia or death. The review of these medical records, were performed in order to comply with a possible underreporting of complete uterine ruptures to the DMBR. Altogether 1839 medical records were reviewed (Fig 1).

Medical records with information on both the current labour and all previous pregnancies were retrieved from various gynaecologic and obstetric departments in Denmark. Data were validated by review of medical records, and only cases with one previous caesarean delivery performed in Denmark, a singleton pregnancy and a complete uterine rupture during TOLAC at term were included. Our intention was to study the uterine ruptures occurring during TOLAC and the not the few ones that occur before onset of labour. In our study, labour was defined as a woman with contractions or induction of labour, but not necessarily having an orificium dilated to 4 cm or more. We did not include women with pre-labour emergency CS without induction or contractions.

Subsequently, controls were identified in the DMBR (1997–2008). For each case two controls were selected as the two subsequent chronological deliveries in the DMBR among all the women with a singleton term pregnancy, a planned TOLAC, an available medical record, and no uterine rupture. Their medical records were requested, and all data were validated as described above. Only controls with one previous caesarean delivery performed in Denmark, a singleton pregnancy, TOLAC at term and no uterine rupture were included. We reviewed 1839 medical records in order to identify and validate cases and included 175. After selection of controls in the DMBR we reviewed further 392 medical records and included 272 controls (Fig 1). The review was performed by D. Thisted and validated by L. Krebs.

Information on pre-pregnancy risk factors and complications during the current and previous pregnancies and deliveries was collected by a thorough manual review of each medical record from cases and controls. The review included the labour with a planned TOLAC as well as all previous labours. The previous caesarean sections in both cases and controls were performed in the time period of 1982 to 2007. In Denmark, the surgeon performing the procedure is responsible for preparing the operative report in the medical record. During the period from 1982 to 2007 medical records were mostly paper-based and operative notes were either; dictated, handwritten or generated from a pre-printed form completed by the surgeon. We obtained the information regarding the surgical technique from manual review of each medical record. If there was no specific information regarding outcome measures such as closure in one or two layers, use of angle sutures or use of locked sutures, data were recorded as missing values. If information regarding birth weight was missing in the medical record we used the information recorded in the DMBR. All data were entered into a database and analysed using STATA 12.1. Data were entered into the database by the corresponding author D. Thisted; L. Krebs performed validation of the database.

Odds ratios (OR) with 95% confidence intervals (95% CI) were calculated by use of chi2 test in marginal two-by-two contingency tables. Adjusted odds ratios (aOR) were estimated using logistic regression analysis in which uterine rupture was the outcome. The explanatory variables were selected based on both the uni-variable analysis and if pre-existing evidence have suggested an association to uterine rupture. Except for uterine closure, in which missing values were treated as a variable, missing values were omitted in the regression analysis.

No experiments on human or nonhuman animals or other species was performed. Reporting of the study followed the STROBE guidelines. This study was approved by the Danish Data Protection Agency ((Journal number: 2008-41-2256), initial approval on May 23, 2008, extended approval on July 21, 2014 (Journal number: 2014-41-3289)), and the Danish Health and Medicines Authority (Journal number: 3-3013-168/1, approval date September 7, 2012).

## Results

Altogether, 175 cases and 272 controls were included in the study. Since both cases and controls had to meet to the above-defined criteria, we were not able to include two controls per case as initially planned (Fig 1). In no cases and in two controls, chromic catgut was used for suturing the uterus. The remaining cases and controls were sutured with a monofilament polyglyconate suture, a polyglycolic acid suture, or a polyglactin suture.

Maternal characteristics in cases and controls are presented in Table 1. Women with uterine rupture were more often non-smokers. Maternal age, height, BMI and ethnicity did not differ between cases and controls (Table 1).

**Table 1:** Maternal characteristics at trial of labour after caesarean (TOLAC) and their association to complete uterine rupture. (Denmark 1997–2008).

Maternal characteristics at TOLAC	Cases	Controls	OR (95% CI)	*P*
	*n* = 175	*n* = 272		
	*n* (%)	*n* (%)		
**Maternal height–cm**				
> 165 cm	88 (50.3)	150 (55.2)	Ref	
<- 165 cm	68 (38.8)	101 (37.1)	1.15 (0.77–1.72)	0.505
Missing data height	19 (10.9)	21 (7.7)		
**Maternal BMI**				
<- 30	130 (74.3)	219 (80.5)	Ref	
> 30	26 (14.9)	31 (11.4)	1.41 (0.80–2.48)	0.229
Missing data BMI	19 (10.8)	22 (8.1)		
**Maternal age–years**				
<- 38	160 (91.4)	243 (89.3)	Ref	
> 38	15 (8.6)	29 (10.7)	0.79 (0.41–1.51)	0.469
**Ethnicity**				
Caucasian	152 (86.8)	244 (89.7)	Ref	
Non-caucasian	22 (12.6)	26 (9.6)	1.36 (0.74–2.48)	0.318
Missing data	1 (0.6)	2 (0.7)		
**Smoking**				
Non-smoking	128 (73.1)	189 (69.5)	Ref	
Smoking	20 (11.5)	55 (20.2)	0.54 (0.31–0.93)	0.028
Missing data–smoking	27 (15.4)	28 (10.3)		

Characteristics from the previous pregnancy and labour in which a caesarean had been performed are presented in Table 2. A uni-variate analysis revealed that use of single layer compared to double layer closure of the uterotomy (OR 1.72 CI:1.12–2.64), absence of angle sutures (OR 2.52 CI:1.48–4.31) and birth weight ≥ 4000g (OR 1.91 CI 1.04–3.48) were associated with an increased risk of uterine rupture. Gestational age at delivery or cervical dilatation at the time of caesarean was not associated with uterine rupture (Table 2). Due to the large number of missing data regarding use of locked sutures in the first layer closure, we were not able to estimate the possible effect on uterine rupture (Table 2).

**Table 2:** Characteristics assessed from the caesarean delivery (CD) prior to the trial of labour after caesarean (TOLAC) and their association to complete uterine rupture. (Denmark 1997–2008).

Obstetric history	Cases	Controls	OR (95% CI)	*P*
(*At CD prior to TOLAC)*	*n* = 175	*n* = 272		
	*n* (%)	*n* (%)		
**Gestational age (GA)**				
37+0–42+0	140 (80.0)	212 (77.9)	Ref	
< 37+0	15 (8.6)	34 (12.5)	0.67 (0.35–1.27)	0.217
> 42+0	19 (10.8)	22 (8.1)	1.31 (0.68–2.50)	0.417
GA missing	1 (0.6)	4 (1.5)		
**Birth weight–grams (g)**				
< 3000g	32 (18.3)	85 (31.3)	Ref	
3000-3900g	108 (61.7)	141 (51.8)	2.03 (1.27–3.27)	0.003
≥ 4000g	33 (18.9)	46 (16.9)	1.91 (1.04–3.48)	0.035
Birth weight missing	2 (1.1)	0		
**Fetal position**				
Vertex	123 (70.3)	176 (64.7)	Ref	
Non-vertex	51 (29.1)	92 (33.8)	0.79 (0.53–1.20)	0.271
Missing data	1 (0.6)	4 (1.5)		
**Induction of labour**				
No induction	131 (74.8)	207 (76.1)		
Induction	39 (22.3)	53 (19.5)	1.16 (0.73–1.86)	0.527
Missing data	5 (2.9)	12 (4.4)		
**Cervical dilatation**				
No dilatation– 0 cm	44 (25.1)	72 (26.5)	Ref	
Cervix dilated 0–5 cm	70 (40.0)	96 (35.3)	1.19 (0.73–1.94)	0.476
Cervix dilated 6–9 cm	38 (21.8)	41 (15.1)	1.52 (0.85–2.71)	0.158
Fully dilated– 10 cm	13 (7.4)	32 (11.7)	0.66 (0.32–1.40)	0.282
Missing cervical dilatation	10 (5.7)	31 (11.4)		
**Mode of delivery**				
Planned CS and CS before active labour	100 (56.4)	159 (58.0)	Ref	
Emergency CS during labour	75 (43.1)	113 (41.7)	1.06 (0.72–1.55)	0.784
**Uterine closure**				
2-layer closure	52 (29.7)	101 (37.1)	Ref	
1-layer closure	101 (57.7)	114 (41.9)	1.72 (1.12–2.64)	0.013
Missing data—layers	22 (12.6)	57 (21.0)	0.75 (0.41–1.36)	0.342
Angle sutures	57 (32.6)	115 (42.3)	Ref	
No angle sutures	45 (25.7)	36 (13.2)	2.52 (1.48–4.31)	< 0.001
Missing data–angle sutures	73 (41.7)	121 (44.5)		
Locked sutures in 1^st^ layer	29 (16.6)	28 (10.3)	Ref	
Unlocked sutures	59 (33.7)	88 (32.4)	0.65 (0.35–1.20)	0.165
Missing data–locked sutures	87 (49.7)	156 (57.3)		

Characteristics from the pregnancy with TOLAC are presented in Table 3. The unadjusted analysis revealed that at least one previous vaginal delivery was associated with a decreased risk of uterine rupture (OR 0.36 CI:0.22–0.57). An inter-delivery interval of more than 24 months reduced the risk of uterine rupture (OR 0.32 CI:0.32–0.62) but no significant association was found considering an inter-delivery interval between 18–24 months. Induction of labour, especially when the cervix was unfavourable, deduced by the use of prostaglandins or double balloon catheter (OR 2.34 CI: 1.48–3.72) increased the risk of uterine rupture. Use of epidural (OR 3.16 CI: 2.10–4.76), augmentation by oxytocin for more than one hour (OR 2.82 CI:1.64–4.85), birth weight ≥ 4000g (OR 2.96 CI 1.26–6.91) and signs of fetal asphyxia, deduced by the need of a fetal scalp pH (OR (1.81 CI: 1.05–3.11) also imposed an increased risk of uterine rupture (Table 3).

**Table 3:** Characteristics assessed from pregnancy and birth with trial of labour after caesarean (TOLAC), and their association to complete uterine rupture. (Denmark 1997–2008).

TOLAC characteristics	Cases	Controls	OR (95% CI)	*P*
	*n* = 175	*n* = 272		
	*n* (%)	*n* (%)		
**Parity** (Vaginal deliveries prior to TOLAC)				
No vaginal deliveries	148 (84.6)	180 (66.2)	Ref	
≥ 1 vaginal delivery	27 (15.4)	92 (33.8)	0.36 (0.22–0.57)	< 0.001
Vaginal delivery after last CD*	12 (6.9)	66 (24.3)	0.22 (0.12–0.41)	< 0.001
Interval from last CD***				
Interval < 18 months	24 (13.7)	14 (5.1)	Ref	
Interval ≥ 18 –< 24 months	25 (14.3)	26 (9.6)	0.56 (0.24–1.32)	0.185
Interval ≥ 24 months	126 (72)	232 (85.3)	0.32 (0.16–0.62)	0.001
**Gestational age (GA)**				
37+0–42+0	158 (90.3)	249 (91.5)	Ref	
> 42+0	17 (9.7)	23 (8.5)	1.16 (0.60–2.25)	0.649
**Birth weight–grams (g)**				
< 3000 g	8 (4.6)	31 (11.4)	Ref	
3000-3999g	114 (65.1)	179 (65.8)	2.47 (1.12–5.45)	0.025
≥ 4000g	45 (25.7)	59 (21.7)	2.96 (1.26–6.91)	0.012
Birth weight missing	8 (4.6)	3 (1.1)		
**Induction of labour**				
No induction	112 (64.0)	216 (79.4)	Ref	
Induction (all methods)	61 (34.9)	53 (19.5)	2.22 (1.45–3.41)	< 0.001
- By Prostaglandins or				
Double Balloon Catheter	51 (29.1)	42 (15.4)	2.34 (1.48–3.72)	< 0.001
- Prostaglandins	41 (23.4)	35 (12.9)	2.26 (1.37–3.72)	0.001
- Double balloon catheter	10 (5.7)	7 (2.6)	2.76 (1.06–7.19)	0.038
- By amniotomy or Oxytocin	10 (5.7)	11 (4.0)	1.75 (0.72–4.22)	0.209
Missing data	2 (1.1)	3 (1.1)		
**Analgesia**				
No Epidural	94 (53.7)	213 (77.5)	Ref	
Epidural	81 (46.3)	58 (22.1)	3.16 (2.10–4.76)	< 0.001
Missing data	0	1 (0.4)		
**Augmentation**				
No augmentation (Oxytocin)	81 (46.3)	184 (67.6)	Ref	
Augmentation	94 (53.7)	87 (32.0)	2.45 (1.66–3.62)	< 0.001
Augmentation: 0–1 hour	16 (9.1)	32 (11.8)	1.14 (0.59–2.19)	0.703
Augmentation: 1–3 hours	36 (20.6)	29 (10.6)	2.82 (1.64–4.85)	< 0.001
Augmentation: > 3 hours	42 (24.0)	26 (9.6)	3.67 (2.15–6.28)	< 0.001
Missing data	0	1 (0.4)		
**Surveillance of labour**				
Continuous CTG	153 (87.4)	205 (75.4)		
No scalp pH	143 (81.7)	242 (88.9)	Ref	
≥ 1 scalp pH	31 (17.7)	29 (10.7)	1.81 (1.05–3.11)	0.032
Missing data	1 (0.6)	0		
**Duration of labour**				
Arrival to birth < 10 hours	89 (50.9)	183 (67.3)	Ref	
Arrival to birth ≥ 10 hours	86 (49.1)	83 (30.5)	2.13 (1.44–3.15)	< 0.001
Missing values (arrival to birth)	0	6 (2.2)		
**Stage I (cervix ≥ 4 cm)** ***	118 (67.4)	255 (93.8)		
Stage I < 6 hours	71 (40.6)	199 (73.2)	Ref	
Stage I ≥ 6 hours	44 (25.1)	44 (16.2)	2.80 (1.72–4.57)	< 0.001
Missing value (Stage I)	3 (1.7)	12 (4.4)		
**Stage II**	62 (35.4)	226 (83.1)		
Stage II < 1 hour	32 (18.3)	161 (59.2)	Ref	
Stage II ≥ 1 hour	27 (15.4)	55 (20.2)	2.47 (1.37–4.45)	0.003
Missing value (Stage II)	3 (1.7)	10 (3.7)		

* CD: Caesarean Delivery

** The interdelivery interval is calculated and reported by months with zero decimals

*** Even though only 67.4% of cases and 93.8% of the controls reached stage I (defined strict as cervix **≥** 4 cm) all women had contractions or had the labour induced.

Two logistic regression analyses were performed. The first analysis, regarding the risk factors to be taken into consideration before the decision of TOLAC, adjusted for previous vaginal deliveries, birth weight at the caesarean prior to TOLAC, inter-delivery interval and uterine closure. The second analysis, regarding the risk factors that can be considered during TOLAC, adjusted for uterine closure, induction of labour by either prostaglandins or double balloon catheter, augmentation for more than 1 hour, use of epidural, time at labour ward, and birth weight.

The adjusted analysis showed that neither single layer closure (1^st^ analysis aOR 1.38 CI:0.88–2.17) (2^nd^ anslysis aOR 1.61 CI:0.99–2.59) nor prolonged labour (aOR 1.07 CI:0.63–1.82) remained significantly related to uterine rupture (Table 4).

**Table 4:** Adjusted odds ratios (aOR) for risk factors for complete uterine rupture from previous and present delivery Denmark 1997–2000.

**Risk factors**	**Cases**	**Controls**	**OR (95% CI)**	***P***	aOR*	***P***
	***n* = 175**	***n* = 272**			**(95% CI)**	
	***n* (%)**	***n* (%)**				
2-layer closure	52 (29.7)	101 (37.1)	Ref			
1-layer closure	101 (57.7)	114 (41.9)	1.72 (1.12–2.64)	0.013	1.38 (0.88–2.17)	0.160
Missing data—layers	22 (12.6)	57 (21.0)	0.75 (0.41–1.36)	0.342	0.65 (0.35–1.21)	0.174
No vaginal deliveries	148 (84.6)	180 (66.2)	Ref			
≥ 1 vaginal delivery	27 (15.4)	92 (33.8)	0.36 (0.22–0.57)	< 0.001	0.41 (0.25–0.68)	0.001
Birth weight < 3000g	32 (18.3)	85 (31.3)	Ref			
Birth weight 3000-3900g	108 (61.7)	141 (51.8)	2.03 (1.27–3.27)	0.003	1.93 (1.18–3.16)	0.009
Birth weight ≥ 4000g	33 (18.9)	46 (16.9)	1.91 (1.04–3.48)	0.035	2.02 (1.08–3.78)	0.028
Interdelivery interval < 18 months	24 (13.7)	14 (5.1)	Ref			
Interdelivery interval ≥ 18 - < 24 months	25 (14.3)	26 (9.6)	0.56 (0.24–1.32)	0.185	0.51 (0.21–1.27)	0.148
Interdelivery interval ≥ 24 months	126 (72.0)	232 (85.3)	0.32 (0.16–0.62)	0.001	0.38 (0.18–0.78)	0.009
**Risk factors**	**Cases**	**Controls**	**OR (95% CI)**	***P***	aOR**	***P***
	***n* = 175**	***n* = 272**			**(95% CI)**	
	***n* (%)**	***n* (%)**				
2-layer closure	52 (29.7)	101 (37.1)	Ref			
1-layer closure	101 (57.7)	114 (41.9)	1.72 (1.12–2.64)	0.013	1.61 (0.99–2.59)	0.052
Missing data—layers	22 (12.6)	57 (21.0)	0.75 (0.41–1.36)	0.342	0.71 (0.37–1.37)	0.310
No induction with unfavorable cervix***	122 (69.7)	227 (83.5)	Ref			
Induction with unfavorable cervix ****	51 (29.1)	42 (15.4)	2.26 (1.43–3.57)	< 0.001	2.10 (1.19–3.71)	0.010
No Epidural	94 (53.7)	213 (77.5)	Ref			
Epidural	81 (46.3)	58 (22.1)	3.16 (2.10–4.76)	< 0.001	2.17 (1.31–3.57)	0.002
No augmentation (Oxytocin) < 1 hour	97 (54.4)	216 (79.4)	Ref			
Augmentation (Oxytocin) > 1 hour	78 (44.6)	55 (20.2)	3.16 (2.09–4.77)	< 0.001	2.03 (1.20–3.44)	0.008
Time from arrival to birth < 10 hours	89 (50.9)	183 (67.3)	Ref			
Time from arrival to birth ≥ 10 hours	86 (49.2)	83 (30.5)	2.13 (1.44–3.15)	< 0.001	1.07 (0.63–1.82)	0.790
Birth weight < 3000g	8 (4.6)	31 (11.4)	Ref			
Birth weight 3000-3900g	114 (65.1)	179 (65.8)	2.47 (1.12–5.45)	0.025	2.49 (1.06–5.85)	0.037
Birth weight ≥ 4000g	45 (25.7)	59 (21.7)	2.96 (1.26–6.91)	0.012	2.65 (1.05–6.64)	0.038

*Adjusted for: previous vaginal deliveries, inter-delivery interval, birth weight at caesarean prior to TOLAC and uterine closure (2 cases were omitted from the analysis due to missing data of birth weight)

** Adjusted for uterine closure, induction, augmentation, use of epidural and time at labour ward (10 cases and 12 controls were omitted from the analysis due to missing data

*** No induction by Prostaglandins or a double balloon catheter.

**** Induction by Prostaglandins or a double balloon catheter

Complications related to the first caesarean delivery such as infection (endometritis, wound infection, or fever), post partum haemorrhage, or placental abnormalities (previa or placenta accrete) were not associated with uterine rupture in a subsequent TOLAC (data not shown). Also, there was no association between previous dilatation and curettage or other previous genital surgery (other than a caesarean delivery) and uterine rupture (data not shown).

Pregnancy complications such as preeclampsia or hypertension in pregnancy and pre-pregnancy medical disorders (diabetes, thyroid disorders, rheumatoid arthritis, asthma, or inflammatory bowel diseases) were not associated with uterine rupture in a subsequent TOLAC (data not shown).

A missing value analysis, in which information on single or double layer closure was regrouped into either “missing” or “non-missing” was performed. We found that information on single or double layer closure were more often missing in women who were non-caucasian, had BMI above 30 or were below 165 cm high.

## Discussion

In the present population-based case-control study we found no significant association between single layer uterine closure and complete uterine rupture in a subsequent TOLAC after adjusting for previous vaginal deliveries, Birth weight ≥ 4000g at previous caesarean, inter-delivery interval, and induction of labour (by either prostaglandins or double balloon catheter), augmentation by oxytocin (for more than one hour), use of epidural actual birth weight ≥ 4000g and prolonged labour.

Induction of labour with an unfavourable cervix, augmentation by oxytocin for more than one hour, high birth weight use of epidural and measurement of fetal scalp pH indicating a cardiotocography with signs of fetal asphyxia, all increased the risk of a uterine rupture. In contrast, at least one previous vaginal delivery reduced the risk of a subsequent uterine rupture. An inter-delivery interval of 24 months or more decreased the risk of uterine rupture.

The strengths of this study are the use of validated data, the number of cases included, the strict definition of uterine rupture, and inclusion of only complete uterine ruptures. Thus we avoided the risk of including the less well-defined partial uterine ruptures [6], which are often without medical complications and incidentally diagnosed at repeat caesarean delivery [2,3].

Previous case-control or cohort studies have investigated the impact of single layer closure and the risk of uterine rupture with conflicting results [11,13,19]. Obviously, a randomized controlled study comparing single-layer to double-layer closure in caesarean delivery would be the gold standard when estimating the impact of the operative technique on the risk of uterine rupture. The CEASAR and CORONIS studies [10, 20] may answer the question; however, as illustrated in the CORONIS study [10] it takes many years of follow-up before the impact of an operative technique on future pregnancies can be estimated. In Denmark, the change in suture technique was a consequence of a change in a national guideline in 1998. The study period 1997–2008 with the previous caesarean sections performed during the period from 1982 to 2007 was selected in order to obtain a material with equal distribution of cesareans with uterine closure in one and two-layer.

In our study information on single or double layer closure was missing in 12.6% of cases and 21% of controls. In order to evaluate this potential bias we repeated the logistic regression analysis with all missing data recoded as single layer closure, which did not change the results. When recoding all missing data as double layer closure we found a shift towards an association between uterine rupture and single layer closure.

The limitations of this study are the large amount of missing data regarding use of angle sutures and locked versus un-locked sutures. A few studies have found an increased risk of uterine rupture when the single layer or the inner layer of a double layer closure is performed using a locked suture [21]. No previous studies have estimated the association between use of angle sutures, defined as the application of one or two single sutures at the uterine angles before closure of the uterotomy, and risk of uterine rupture in a subsequent TOLAC. Most bleeding takes place from the angles of the incision. Applying an angle suture should, in theory, secure both haemostasis and sufficient closure of the lateral angle of the uterotomy. In the operative technique introduced in Denmark in 1998 neither use of angle sutures nor use of locked or unlocked sutures was addressed [9]. Thus, the use or non-use of angle sutures or locked sutures was not routinely addressed in the operative record, and un-recorded angle sutures or locked sutures can disguise the fact that they have actually been there. With regard to information on both angle sutures and locked sutures, the present study is underpowered to make a valid conclusion.

Our results are in agreement with a Swedish cohort study [15] and a recent meta-analysis [14] that concluded that single-layer closure was not a risk factor for uterine rupture. Performing a caesarean involves a number of different elements. We cannot exclude that other aspects of suturing the uterotomy could explain our findings [22]. During the same period as the new technique for caesarean delivery was recommended in Denmark many obstetricians changed to a practice where suturing of the uterotomy included the full uterine wall in contrast to the previous routine where inclusion of the endometrium was avoided as far as possible [9].

Augmentation by oxytocin is a well-known risk factor for uterine rupture. Cahill et al [23] found that the risk of uterine rupture was proportional to both the duration and the dose of oxytocin administered. Also, induction of labour is a well known risk factor for uterine rupture [1,24,25]. Studsgaard et al. found an association between induction of labour by a double balloon catheter and uterine rupture. However, the risk was not found significantly increased [25]. While, the same study found a significant association between uterine rupture and use of epidural for analgesia (OR 2.2, CI 1.1–4.9) [25], Weimer et al (a nested case-control study) found no association between uterine rupture and use of epidural [26]. Since the use of epidural has no impact on the strength of the uterine wall, the need for epidural, especially when used in combination with augmentation by oxytocin, should be considered as an indicator of labour dystocia. Consequently, the lack of significance of prolonged labour in the adjusted analysis (Table 4) should be interpreted with caution.

Previously Bujold et al [27] found that an inter-delivery interval of less than 18 months increased the risk of uterine rupture. In our study we found that compared to an inter-delivery interval of 18 months, an interval between a caesarean delivery and a TOLAC of at least 24 months decreased the risk of uterine rupture.

In this study single-layer uterine closure did not remain significantly associated to uterine rupture during TOLAC at term after adjustment of confounding factors. Still, induction of labour with an unfavourable cervix, Birth weight ≥ 4000g and indicators of prolonged labour, such as use of epidural and augmentation by oxytocin for more than 1 hour, are major risk factors for uterine rupture.
